# Editorials

**Published:** 1891-05

**Authors:** 


### HOMŒOPATHIC PHYSICIAN,

A MONTHLY JOURNAL OF

HOMŒOPATHIC MATERIA MEDICA AND CLINICAL MEDICINE.

"If our school ever gives up the strict inductive method of Hahnemann, we are lost, and deserve only to be mentioned as a caricature in the history of medicine."—CONSTANTINE HERING.

Vol. XI. MAY, 1891. No. 5.

### EDITORIALS.

KOCH'S LYMPH AND SWAN'S TUBERCULINUM.—The genius of Hahnemann enabled him to give to the world, beside the law of cure, an unerring insight into the nature of disease. All that has been discovered by both pathological and physiological research since his time goes to prove the truths he proclaimed. With a knowledge of the teachings of this great philosopher one possesses the ability to pass correct judgment on all that is offered belonging to the sphere of disease and its cure.

Thus when Koch heralded to the world his great and new (?) discovery we did not hesitate to place on record views which are in antagonism to the claims made for his remedy.

We have continued to give careful attention to all that has been offered in its favor by Koch and his adherents, and we are still of the opinion that it is of no value as a remedy for tuberculosis *per se*, but in isolated cases, if properly administered, it may effect some good. In other words, it is but one remedy, and can only be of service after it has been proved upon healthy individuals, and thus only can its curative sphere be known.

Mortality tables are now showing the harm that results from using indiscriminately a substance which has such pathogenetic power that all nosodes are now known to possess.

(We need not stop here to remark the illogical position of those scientists (?) who are so opposed to the use of secret remedies, and yet who flocked to Berlin to learn of the grand discovery. The past two months have shown that so-called scientific medicine is as far as ever from a sound therapy.)

Even two months have sufficed to show the weakness of the claims made for this remedy, and if the number of deaths thus far caused by it be not sufficient to prove how hurtful it is, and to how little confidence it is entitled, we have the testimony of some of the leading allopathic physicians of Berlin to offer. On the 21st ult. the subject was under discussion in the Berlin Medical Society. Virchow showed specimens from the body of a man who had been admitted to the hospital for pleurisy with effusion. He remained " in a satisfactory state " until injections with Koch's lymph were begun, of which five were given, each of five milligrammes. "He died, and the examination made showed, in addition to old induration at both apices and the remains of pleurisy, a widespread miliary tuberculosis in lungs, spleen, kidneys, and liver."

Other post-mortems have shown the power of the remedy to convert a local tubercular condition into general tuberculosis. Dr. Ewald, after many experiments on one hundred and fourteen cases treated at the Augusta Hospital, affirmed that "*he had not yet seen one case which he could say was cured.*" He further said : "According to present experience the physician is in the position of an operator who cannot foretell the issue of a difficult operation. The patient must be told that the remedy may produce the most severe effects, and even cause a fatal result." Summing up, he said the conviction was borne in on him that "*in no single case*, with the exception, perhaps, of a few in the very earliest stages, and then not with positive certainty, *can one say how the case will go*, either in respect to the character of the reactions or to the ultimate result."

Contrast this with Hahnemann's directions in respect of finding the curative powers of a remedy ! On the one hand, we have a hazardous, death-causing mode, on the other a law of nature by which the true physician is able to conquer disease without jeopardizing the health of a patient.

Induction, that process of drawing a general conclusion from particular cases, will, in a measure, render the Hahnemannian competent to approximate the curative value of such substances as nosodes. This has been demonstrated by Dr. Samuel Swan, of New York, who some twelve years ago published cures made by *Tuberculinum* before one proving was made. These cases are of so much interest now that we offer no apology for again calling attention to them. They are to be found in the *Organon*, Vol. II, 1879, page 342.

Case by Dr. Swan: "Lizzie R. came to my clinic on the 23d of July, 1874, complaining of cough, with pain and soreness of the chest; violent palpitation of the heart, with sharp pains through it in various directions; the beats of the heart were very violent, but not very fast; pulse 100; severe pains in the kidneys; urine very dark but clear; constipation; burning pain the whole length of the spine; pain in apex of both lungs; frequent attacks of hoarseness, with sometimes entire loss of voice, without adequate cause; rheumatic pains across the back from one shoulder to the other; menstruation regular as to time, but painful and scanty; some leucorrhœa; pain in right ovary; constant headache from one parietal protuberance to the other, around the front of the head.

"The history of the case showed that at the age of three years she was frightened into a fit; this settled down to chorea, which continued until she was thirteen."

She was twenty-one when Dr. Swan first saw her. From November, 1874, she had various symptoms—aphonia, pain in kidneys, spasm of the chest, which prevented inspiration for some time, when the spasm relaxed, the lungs became inflated, and she was unable to expire till she became unconscious.

"From the 19th of May till September 13th, 1875, she had severe attacks of pain in kidneys, pain and weakness of entire spine, and constant headache. From this time to January, 1877, she was confined to her bed—nearly sixteen months—and during that time passed through a variety of conditions. For nine months of the sixteen the only nourishment she took was chocolate ice-cream. In November, 1875, she then lying speechless, but able to hear and see perfectly, the upper lip began to thicken and curl up, till at last the vermilion border was turned tightly against her nose. The lower lip during this time was drawn tightly over the lower teeth, the upper edge drawn over the corners, the upper teeth shut down tightly on the lip, with complete trismus, the muscles of the cheek being, on pressure, not distinguishable from bone. During this time she was nourished by milk given through an aperture made by the loss of a front tooth, and even then, so rigid was the throat, it was with the greatest difficulty that she could swallow.

" This rigidity suddenly ceased on January 25th, 1876, to be followed by a very sore mouth, the whole lining of the roof and cheeks peeling off in great sheets, the gums ulcerated, the teeth loose, and an intense fetid odor from the mouth. About the middle of February this rapidly healed, and the left arm became paralyzed; February 25th the headache increased in intensity until she became unconscious, and had a spasm on the evening of the 26th until three A. M. on the 27th. These spasms continued to occur daily for nine weeks. The spasm was a rapid vibration of head and body to the hips, the arms being rigid, but trembling. During the attack great loquacity, talking of the persons and occurrences of the day, making fun of everything. On April 28th these ceased and she had a severe cough for a week or two. On May 27th she began screaming with headache, became unconscious, and began beating up and down with her right arm from the elbow, the left arm being paralyzed. (She described this pain in the head 'as if the brain in front were red hot.') In this unconscious state she commenced a series of very violent but automatic movements, such as boring her head in the bed, while the body simulated the movements of a serpent wounded in the head, endeavoring to burrow into the ground, at other times bounding round as a chicken does with its head cut off, requiring several persons to prevent her from dashing her head against the wall or floor. These performances lasted about an hour; first one each day, increasing to five each day, commencing at six A. M. and ceasing at five P. M.; during the night she slept quietly.

These gradually decreased till they came with regularity every third day, then every fifth day, then every seventh day, and so continued for about sixteen weeks. * * * From the time these ceased, in the latter part of September, 1876, she was confined to her bed with extreme sensibility of the spine, till Christmas, when she began to improve and sit up free from all complaint, except the ever-present headache. About March 1st a severe cough commenced, increasing in intensity, with sweet-tasting, purulent expectoration, great emaciation, profuse debilitating night-sweats; great prostration, and she appeared to be in the last stage of consumption. This continued six or eight weeks, then suddenly ceased, and in a few days she was wonderfully changed for the better, being up and apparently well.

"On May 31st, 1877, a new phase appeared. While sewing or talking she would become suddenly unconscious, then began screaming, tearing her hair, beating her head with her fists, or trying to dash it against the wall or floor. These continued daily until July, when the spasm with the vibratory motion commenced, with rolling of the head from side to side, and moaning. These continued five weeks, then the unconscious fits, with screaming, tearing the hair, and beating the head, returned, coming at least twice a week, and continuing till November 18th, when she said she would have an attack. I inquired what were the premonitory symptoms she then noticed. She said that a few hours before an attack she would have *a shuddering like a chill*, that seemed to go from her brain down her spine.

"Heretofore she could never help me with symptoms, being free from all complaint between the attacks, except sometimes fatigue and the ever-present headache, which was always in front. When asked about an attack she said that the head would suddenly seem to swell over the eyes and the pain become 'horrid,' and she knew no more.

"This shuddering like a chill being so like the formation of pus, I gave Tuberculinum$^{mm}$ (Swan), one dose. [Dr. Swan's Tuberculinum is made of pus from a pulmonary abscess.]

"That evening she had an attack of great violence, lasting nearly two hours, *and that is the last she had.* Twice during the subsequent week they commenced, then ceased. On the following 25th of November, 1877, she had a second dose; on December 2d the third dose; December 9th the fourth dose to take whenever she had any premonitory symptoms, but she has had no occasion to use it. About December 3d a most profuse purulent leucorrhœa set in, flowing so freely as to require four or five napkins a day, and continued till the middle of January with more or less flow.

"Her mother was taken sick in January, 1878, and my patient was able to do all the housework and relieve her mother of all care. She and her family consider her well, as for the first time in her life since she can remember she has been entirely free from headache, and this for some weeks. She still feels weak on going up-stairs, with dyspnœa and palpitation of heart. In December, 1878, the patient got frightened by a fire in the house and fainted, or had a convulsion. Since then her head has ached and old spasms have returned, but under Anthrac. she is beginning to improve, the headache having ceased, but leaving a number of other curious symptoms."

Another case is reported by Dr. Biegler, of Rochester, in which Tuberculinum, which was advised and sent by Dr. Swan, cured a child six years old of an attack of tubercular meningitis after two old-school doctors had pronounced the case hopeless.

These two cases show what Tuberculinum is capable of doing, but we must not rest here. Until we have a thorough proving of this remedy we shall not be able to fix its place specifically. It is incumbent upon us to prove not only this but other nosodes, and then we shall be competent to say to what condition of disease they are scientifically applicable. G. H. C.

---

Government Indorsement of Professor Koch.—Before the first craze over Koch's claims had subsided, we saw it suggested that Congress appropriate a large sum of money for the purchase of the lymph.

It is to be hoped, since it has become known that the claims cannot be made good, that we shall hear no more of an attempt to throw away the public money, and that our law-makers may be as wise as the following shows the English to be. (This extract was translated by a contributor): G. H. C.

From *La Semaine Médicale*, 4 Février, '91. Translated by Frederic Preston, M. D.:

Thursday last, in the British House of Commons, Colonel Nolan asked the First Lord of the Treasury if the Government of Great Britain would not consider it well to have an understanding with other governments of civilized countries with the view of according to Dr. Koch a pecuniary recompense for the eminent services which he has rendered to humanity.

Dr. Tanner took the floor and expressed himself as follows: "Before the First Lord of the Treasury replies to the question propounded to him by Mr. Nolan, I would desire to know if our colleague has taken into consideration the extraordinarily large number of deaths which have occurred among patients to whom the pretended discovery has been applied?"

Mr. W. H. Smith, Treasurer, then took the floor and made the following declaration:

"I am convinced that Mr. Nolan does not expect to hear me dilate on this subject. For my part, I appreciate fully the generous sentiment which has caused the honorable member to address me his question; but, without depreciating in the least the very great services rendered to humanity by Dr. Koch, one should in the meantime observe that he is not *the* unique savant who has patiently and laboriously searched the resources of nature for the benefit of humanity. [Laughter.] His great recompense is the certain appreciation of the value of his work by the physicians of the entire world [laughter] and the sentiment of having been the benefactor of his kind. [Hear! Hear!] I do not believe that any intervention whatever by the government of the Queen could really augment that satisfaction which Dr. Koch should feel, in presence of the reception of his discovery by the civilized world. Therefore, I hope to be excused from adding a new burden to the Government." [Hear! Hear! and laughter.]

From the New York *Medical Journal*, of February 14th, we take the following. We offer it to show how scientifically allopathy can kill:

A FAILURE WITH KOCH'S REMEDY.

A significant case was reported at the last meeting of the New York Pathological Society, on Wednesday evening, that seems to us to go far to exemplify the force of the cautions inculcated by Virchow's observations, subsequently reinforced by Henoch's, as to the possibility of spreading or intensifying a moderate tubercular invasion by the employment of the Koch treatment. The case was that of a man presenting the rational and physical signs of pulmonary tuberculosis, but in whom no pulmonary cavity could be detected. After he had been given twenty-four injections of Koch's liquid, in the usual doses and at the usual intervals, the number of bacilli in the sputum was found to have increased, and the patient's condition was decidedly worse. A cavity was detected in the apex of the lung, and the patient died shortly after the discontinuance of the Koch treatment. After death, the cavity was found in the lung, about as large as a lemon, and there was miliary tuberculous disease of the lungs. The tuberculous foci were surrounded by intense congestion, and the meninges of the brain and various organs were also highly congested. The opinion was expressed that at the outset the case was eminently a proper one for testing the efficacy of the Koch treatment.

Dr. Benjamin Ward Richardson thus gives vent to his disgust with Koch's sometime secret remedy:

"The fates have not been propitious. The secret is, partly, out, but many believers in it, whilst it was a secret, shrug their shoulders now and think without utterance. Ah! if they had but known that the remedy was a poison, administered in infinitesimal proportions, they would have left it for the homœopaths to manipulate, according to their dogma and their heresy! And here are the homœopaths laughing actually at us of the school of legitimate [sic] physic, because we have been caught vulgarly swallowing their dogma, admitting even the effect of the infinitesimal dose, and they themselves keeping out of all danger within their own lines. Incredible humiliation!"

Our lines are based upon law, and this law not only enables us to keep out of danger, but it permits us to keep our patients away from the danger-line. We shall continue to laugh so long as "the school of legitmate (?) physic" continues on the danger-line. G. H. C.

